# Supplementary material for: Whole-Genome Sequence and Characterization of Ralstonia solanacearum MLY102 Isolated from Infected Tobacco Stalks
Source: Genes (Basel). 2024 Nov 15;15(11):1473. doi: 10.3390/genes15111473 (PMC11593729; doi:10.3390/genes15111473)
Supplement: Supplementary file 1 [file genes-15-01473-s001.zip › genes-3305771-supplementary.pdf]

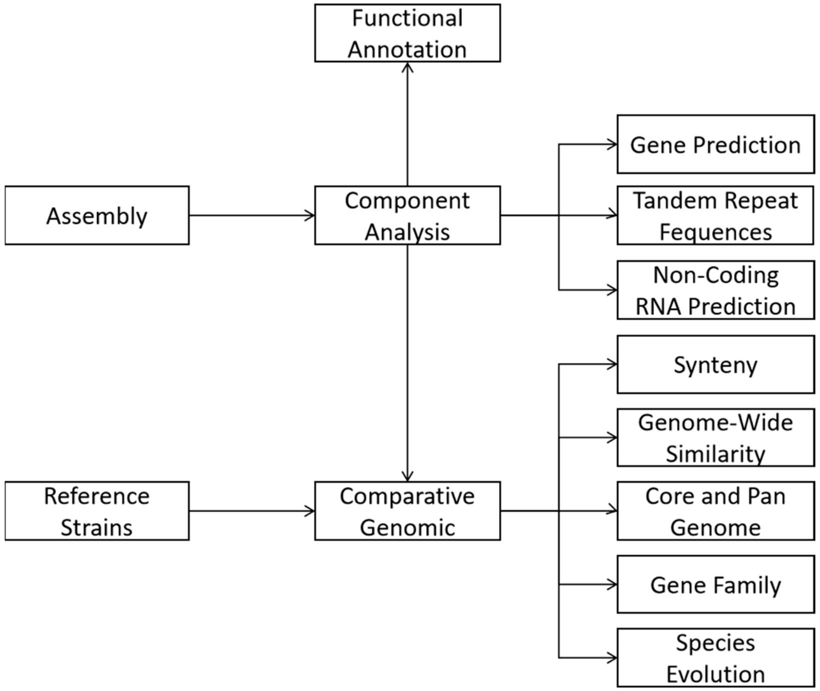

Figure S1. Bioinformatics Analysis Flowchart

Table S1. Synteny rates at nucleic acid level

	RS-SL2064	RS-Rs5	RS-T95	CFBP2957	CMR15	GMI1000	PSI07	Po82	RS-UW251	RS-CIAT078
MLY102	82.023	78.64	82.052	76.579	86.692	93.375	82.011	78.71	77.937	78.557
pMLY102	64.673	57.206	64.673	1.502	83.532	93.402	66.283	60.412	62.002	59.706

Table S2. Synteny statistics at amino acid level between MLY102 and reference strains

	RS-SL2064	RS-Rs5	RS-T95	CFBP2957	CMR15	GMI1000	PSI07	Po82	RS-UW251	RS-CIAT078
Aligned	3884	3721	3892	2666	4120	4358	3881	3817	3838	3696
Target Genes	5283	5283	5283	5283	5283	5283	5283	5283	5283	5283
Target Perc(%)	73.52	70.43	73.67	50.46	77.99	82.49	73.46	72.25	72.65	69.96
Query Genes	4719	4560	4729	3115	6782	4996	4753	4649	4758	4548
Query Perc(%)	82.31	81.6	82.3	85.59	60.75	87.23	81.65	82.1	80.66	81.27
Identity Mean	92.5	91.11	92.51	92.26	95.49	98.79	92.46	91.14	91.15	91.08
Identity Median	94.6	93.42	94.6	94.25	97.58	99.66	94.6	93.52	93.475	93.49
